# Values of debulking surgery for unresectable well-differentiated metastatic pancreatic neuroendocrine tumors: a comparative study

**DOI:** 10.1093/gastro/goad010

**Published:** 2023-03-08

**Authors:** Xi-Tai Huang, Jin-Zhao Xie, Liu-Hua Chen, Jian-Peng Cai, Wei Chen, Li-Jian Liang, Ning Zhang, Xiao-Yu Yin

**Affiliations:** Department of Pancreato-Biliary Surgery, The First Affiliated Hospital, Sun Yat-sen University, Guangzhou, Guangdong, P. R. China; Department of Pancreato-Biliary Surgery, The First Affiliated Hospital, Sun Yat-sen University, Guangzhou, Guangdong, P. R. China; Department of Pancreato-Biliary Surgery, The First Affiliated Hospital, Sun Yat-sen University, Guangzhou, Guangdong, P. R. China; Department of Pancreato-Biliary Surgery, The First Affiliated Hospital, Sun Yat-sen University, Guangzhou, Guangdong, P. R. China; Department of Pancreato-Biliary Surgery, The First Affiliated Hospital, Sun Yat-sen University, Guangzhou, Guangdong, P. R. China; Department of Pancreato-Biliary Surgery, The First Affiliated Hospital, Sun Yat-sen University, Guangzhou, Guangdong, P. R. China; Department of Gastroenterology, The First Affiliated Hospital, Sun Yat-sen University, Guangzhou, Guangdong, P. R. China; Department of Pancreato-Biliary Surgery, The First Affiliated Hospital, Sun Yat-sen University, Guangzhou, Guangdong, P. R. China

**Keywords:** pancreatic neuroendocrine tumor, metastasis, debulking surgery, prognosis

## Abstract

**Background and objective:**

The value of debulking surgery for unresectable well-differentiated metastatic pancreatic neuroendocrine tumor (m-PNET) remains poorly defined. This study aimed to evaluate the outcomes of m-PNET following debulking surgery in our institute.

**Methods:**

Patients with well-differentiated m-PNET in our hospital between February 2014 and March 2022 were collected. Clinicopathological and long-term outcomes of patients treated with radical resection, debulking surgery, and conservative therapy were compared retrospectively.

**Results:**

Fifty-three patients with well-differentiated m-PNET were reviewed, including 47 patients with unresectable m-PNET (debulking surgery, 25; conservative therapy, 22) and 6 patients with resectable m-PNET (radical resection). Patients undergoing debulking surgery had a post-operative Clavien–Dindo ≥ III complication rate of 16.0% without mortality. The 5-year overall survival (OS) rate of patients treated with debulking surgery was significantly higher than that of those treated with conservative therapy alone (87.5% vs 37.8%, log-rank *P *=* *0.022). Besides, the 5-year OS rate of patients treated with debulking surgery was comparable to that of patients with resectable m-PNET undergoing radical resection (87.5% vs 100%, log-rank *P *=* *0.724).

**Conclusions:**

Patients with unresectable well-differentiated m-PNET who underwent resection had better long-term outcomes than those who received conservative therapy alone. The 5-year OS of patients undergoing debulking surgery and radical resection were comparable. Debulking surgery could be considered for patients with unresectable well-differentiated m-PNET if no contraindication exists.

## Introduction

Pancreatic neuroendocrine tumors (PNETs) are rare tumors of the gastrointestinal tract with relative indolent biological behaviors, which comprise 2%–7% of all pancreatic tumors [1, 2]. The majority of patients are found accidentally and usually present advanced diseases in the late course of disease, especially in those with non-functioning-PNET (NF-PNET) [3]. It is estimated that >60% of pNET had distant metastases and 20% of pNET were presented with locally advanced diseases [3–7]. Nevertheless, patients with stage IV PNET still have a 5-year survival rate of ≤60% [8]. Because of the long-term survival potential of patients with advanced disease, the optimal treatment of metastatic PNET (m-PNET) remains controversial.

Provided that radical resection remains the treatment of choice for resectable PNET [9], patients with m-PNET were commonly managed conservatively with drugs, including somatostatin analogs (SSAs), molecular target therapy (everolimus, sunitinib, surufatinib), chemotherapy (capecitabine/temozolomide), and 177Lu-DOTATATE treatment, and/or trans-hepatic arterial embolization (TAE) [10]. However, the primary pancreatic tumor may lead to life-threatening consequences, such as regional portal hypertension and subsequent upper gastrointestinal bleeding, and gastrointestinal obstruction. Whether palliative pancreatectomy of the primary pancreatic tumors is beneficial for the m-PNET elicits strong controversies. Previous studies have found that palliative resection of the primary tumor was associated with a survival benefit in patients with stage IV NF-PNET by analysing the Surveillance Epidemiology and End Results database but failed to analyse post-operative morbidity and mortality due to the lack of important clinicopathological factors and surgical details [11]. Besides, the survival benefits of debulking surgery for liver metastases of PNET has also been demonstrated [6, 12] but failed to be compared with the conservative therapy. Due to the high risk of morbidity and mortality related to pancreatic surgery, aggressive pancreatectomy like pancreatoduodenectomy should be carefully considered for m-PNET [13].

Given the lack of evidence regarding the prognosis after debulking surgery for well-differentiated m-PNET, the present study attempted to evaluate the short-term and long-term outcomes of patients with m-PNET who underwent palliative pancreatectomy in our institute.

## Patients and methods

### Patient selection

Patients with well-differentiated metastatic PNET who were treated between February 2014 and March 2022 at the First Affiliated Hospital of Sun Yat-sen University (Guangzhou, China) were included. The inclusion criterium was that patients were pathologically confirmed as having well-differentiated PNET with distant metastases. The exclusion criteria were (1) presence of other malignancies or (2) pathologically diagnosed as having poorly differentiated pancreatic neuroendocrine carcinoma. This study was approved by the Ethics Committee of our hospital, Guangzhou, China (Approval Number: [2022]495).

### Data collection and definition

Clinicopathological data were retrospectively collected, including preoperative imaging findings, tumor characteristics, treatment course (including conservative therapy, surgical details), and post-operative course.

Distant metastasis was diagnosed based on imaging findings or pathological test. Tumor functionality was evaluated according to the presence of a detectable elevated serum level of the relevant hormone associated with a clinical syndrome. Tumor grade was defined according to the definition of World Health Organization (WHO) grade system.

Resectability of pNET with liver metastases (LM) depends on two aspects, i.e. primary pancreatic tumor and LM. The resectability of the primary pancreatic tumor was defined by the same criteria as those used for pancreatic carcinoma, i.e. those with encasement of superior mesenteric artery and/or celiac axis (CA) and/or common hepatic artery over 180 degrees as well as those with superior mesenteric vein occlusion were considered unresectable. On the other hand, LM of pNET were classified into three types [14]. Type I was defined as a single metastasis regardless of size, type II was defined as an isolated metastatic bulk accompanied by smaller deposits, and type III was defined as a disseminated metastatic spread in the whole liver. Most Type I and part of type II LM were resectable, and type III LM were unresectable. As a whole, the resectable pNET with LM was defined as a resectable primary pancreatic tumor with resectable type I or type II LM. Unresectable disease was defined as unresectable primary pancreatic tumor with resectable or unresectable LM, resectable primary pancreatic tumor with unresectable LM, or extrahepatic metastases.

Debulking surgery referred to the removal of primary pancreatic tumors with/without metastasectomy in technically unresectable disease. Radical resection referred to the removal of primary pancreatic tumors and all metastases in technically resectable disease, confirmed by post-operative radiological examination. Conservative therapy included single, or combined, or sequential administration of somatostatin analogs (SSAs), molecular targeted therapy, cytotoxic chemotherapy, and trans-hepatic arterial embolization.

The level of drainage fluid amylase was tested on post-operative Days 1, 3, 5, and 7. Post-operative complication was evaluated according to the Clavien–Dindo classification [15]. The definition of post-operative pancreatic fistula (POPF) was determined according to the 2016 International Study Group of pancreatic surgery (ISGPS) definition and grading of post-operative pancreatic fistula [16]. Grade B and grade C POPF were defined as clinically relevant POPF (CRPOPF).

All patients were followed up until death or censored at the cut-off date of April 2022. The outcome measured was overall survival (OS). OS was calculated from the date of diagnosis (for patients treated with conservative therapy) or surgery (for patients treated with surgery) to the date of death or the last follow-up.

### Statistical analysis

All statistical analyses were performed by using SPSS version 24.0 software (IBM, Inc., Armonk, NY, USA) and R version 4.0.0 (http://www.Rproject.org). Categorical variables are presented as frequencies with percentages, whereas continuous variables are presented as medians with interquartile range (IQR). Differences between categorical variables were compared by using chi-square test or Fisher’s exact test. Differences between continuous variables were compared by using the Mann–Whitney *U* test. Kaplan–Meier curve and log-rank test were used to compare the differences in survival. A Cox proportional hazard model was used to determine the independent prognostic factors in OS. Two-tailed *P *<* *0.05 was considered statistically significant.

## Results

### Clinicopathological features of patients with unresectable well-differentiated metastatic PNET

**Figure 1.**
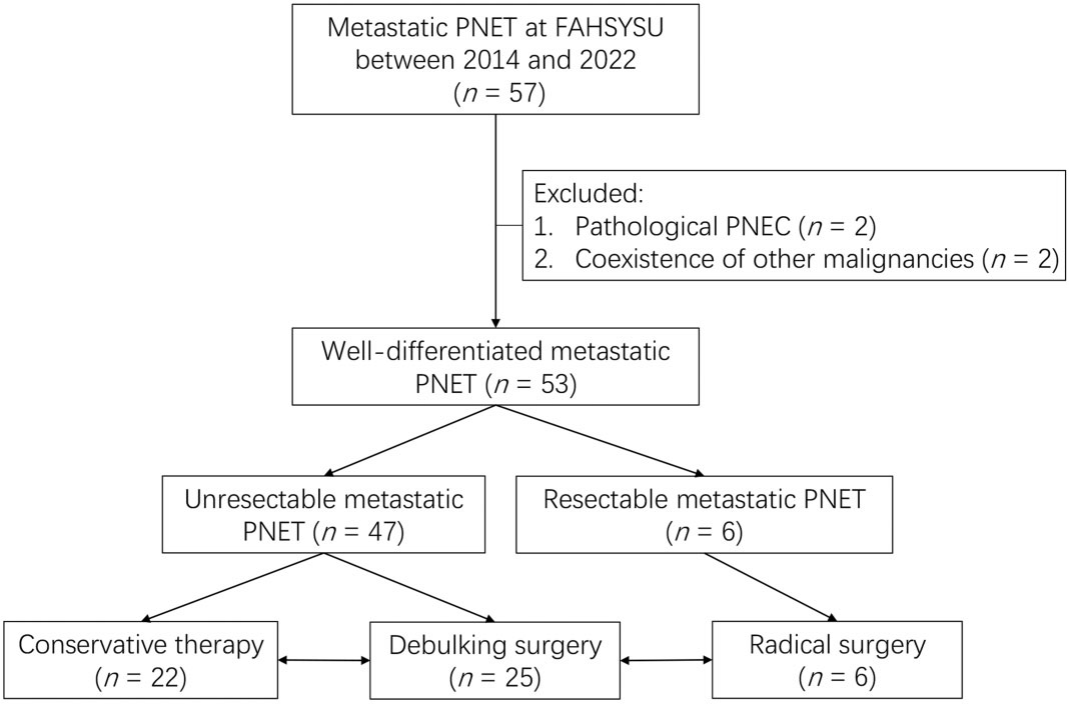
Flow chart of patient assignment in this study. PNET, pancreatic neuroendocrine tumor; FAHSYSU, First Affiliated Hospital, Sun Yat-sen University.

We reviewed and included 53 patients with well-differentiated m-PNET in the present study, including 47 patients with technically unresectable m-PNET and 6 patients with technically resectable m-PNET (Figure 1).

Among unresectable m-PNET patients, 25 patients were treated with debulking surgery, whereas the remaining 22 patients were treated with conservative therapy alone (Table 1). LM were present in all patients with m-PNET. There were no significant differences in the type and burden of LM between the debulking surgery group and the conservative therapy group (Supplementary Table 1). The proportions of patients with symptomatic disease (56.0% vs 86.4%, *P *=* *0.029) and functional PNET (24.0% vs 54.5%, *P *=* *0.032) were lower in the debulking surgery group than in the conservative therapy group. Patients in the debulking surgery group had larger tumors (4.9 vs 2.9 cm, *P *=* *0.011) and more advanced T stage (80.0% vs 45.5%, *P *=* *0.014), but lower rates of lymph node metastasis (32.0% vs 68.2%, *P *=* *0.013) than those in the conservative therapy group. In addition, there were no significant differences in other clinicopathological characteristics between the two groups, including age, sex, body mass index (BMI), co-morbidities, location of the primary pancreatic tumor, or WHO tumor grade.

**Table 1.** Comparison of clinicopathological characteristics of patients with unresectable well-differentiated metastatic PNET treated with debulking surgery and conservative therapy

Feature	Radical resection	Debulking surgery	Conservative therapy	*P*-value^a^	*P*-value^b^
	(*n *=* *6)	(*n *=* *25)	(*n *=* *22)		
Median age (range), years	48 (39–57)	49 (42–59)	41 (35–52)	0.751^c^	0.348^c^
Female, *n* (%)	3 (50.0%)	16 (64.0%)	11 (50.0%)	0.653^d^	0.333^e^
BMI, kg/m^2^	22.5 (20.1–23.8)	21.1 (19.6–22.7)	22.1 (20.2–25.0)	0.314^c^	0.073^c^
Diabetes, *n* (%)	1 (16.7%)	4 (16.0%)	2 (9.1%)	1.000^d^	0.670^d^
Hypertension, *n* (%)	2 (33.3%)	5 (20.0%)	3 (13.6%)	0.596^d^	0.706^d^
Symptomatic disease, *n* (%)	6 (100%)	14 (56.0%)	19 (86.4%)	0.066^d^	0.029^d^
Functional status, *n* (%)	1 (16.7%)	6 (24.0%)	12 (54.5%)	1.000^d^	0.032^e^
Histology, *n* (%)				1.000^d^	0.008^d^
Non-functioning PNET	5 (83.3%)	19 (76.0%)	10 (45.5%)		
Insulinoma	0 (0%)	1 (4.0%)	5 (22.7%)		
Gastrinoma	0 (0%)	2 (8.0%)	7 (31.8%)		
Others	1 (16.7%)	3 (12.0%)	0 (0%)		
Primary tumor size, cm	3.0 (2.2–3.6)	4.9 (3.6–6.5)	2.9 (2.4–5.4)	0.004^c^	0.011^c^
Primary tumor location, *n* (%)				1.000^d^	0.526^e^
Head and neck	2 (33.3%)	8 (32.0%)	9 (40.9%)		
Body and tail	4 (66.7%)	17 (68.0%)	13 (59.1%)		
AJCC T stage, *n* (%)				0.001^d^	0.014^e^
T1+T2	6 (100%)	5 (20.0%)	12 (54.5%)		
T3+T4	0 (0%)	20 (80.0%)	10 (45.5%)		
WHO grade, *n* (%)				1.000^d^	0.793^d^
Grade 1	1 (16.7%)	6 (24.0%)	3 (13.6%)		
Grade 2	5 (83.3%)	17 (68.0%)	17 (77.3%)		
Grade 3	0 (0%)	2 (8.0%)	2 (9.1%)		
Lymph node metastasis, *n* (%)	2 (33.3%)	8 (32.0%)	15 (68.2%)	1.000^d^	0.013^e^
AJCC M stage, *n* (%)				0.553^d^	1.000^d^
M1a	6 (100%)	20 (80.0%)	18 (81.8%)		
M1c	0 (0%)	5 (20.0%)	4 (18.2%)		

a Comparison between the radical resection group and the debulking surgery group.

b Comparison between the debulking surgery group and the conservative therapy group.

c Mann–Whitney *U* test.

d Fisher’s exact test.

e Chi-square test.

PNET, pancreatic neuroendocrine tumor; BMI, body mass index; AJCC, American Joint Committee on Cancer; WHO, World Health Organization.

All patients undergoing debulking surgery were given adjuvant therapy (Supplementary Table 2). The one-, two-, and three-line therapies for patients who received conservative therapy are presented in Supplementary Table 3.

### Short-term outcomes of patients with well-differentiated metastatic PNET who underwent surgery

The surgical details and short-term outcomes of patients with m-PNET treated with radical resection and debulking surgery were compared (Table 2). The minimally invasive rate was 33.3% (2/6) and 56.0% (14/25) in the radical resection group and debulking surgery group, respectively. In the debulking surgery group, most patients (17/25, 68.0%) underwent distal pancreatectomy. Ten patients (40.0%) underwent combined organ resection, including the liver, stomach, colon, and left adrenal gland. The clinically relevant POPF rate was 12.0% (grade B leakage: 3/25, 12.0%; no grade C leakage). The post-operative major complication (Clavien–Dindo ≥ III) was 16.0% (4/25) with the median post-operative length of stay of 10 (IQR, 9–15) days. No death occurred in patients undergoing debulking resection.

**Table 2.** Comparison of operative details and post-operative outcomes between patients with well-differentiated metastatic PNET treated with radical resection and debulking surgery

Feature	Radical resection (*n *=* *6)	Debulking surgery (*n *=* *25)	*P*-value^a^
ASA classification			0.634
I–II	4 (66.7%)	19 (76.0%)	
III–IV	2 (33.3%)	6 (24.0%)	
Type of pancreatic surgery			1.000
Pancreatoduodenectomy	2 (33.3%)	6 (24.0%)	
Distal pancreatectomy	4 (66.7%)	17 (68.0%)	
Others	0 (0%)	2 (8.0%)	
Surgery approach			0.394
Open surgery	4 (66.7%)	11 (44.0%)	
Robotic-assisted surgery	2 (33.3%)	14 (56.0%)	
Operative time, min	375 (298–610)	375 (290–430)	0.608^b^
Intraoperative blood loss, mL	350 (100–850)	150 (50–300)	0.105^b^
Blood transfusion	1 (16.7%)	5 (20.0%)	1.000
Additional organ resection	6 (100.0%)	10 (40.0%)	0.018
CRPOPF	0 (0%)	3 (12.0%)	1.000
Clavien–Dindo ≥ III complication	1 (16.7%)	4 (16.0%)	1.000
Mortality	0 (0%)	0 (0%)	NA
Post-operative length of stay, days	13 (11–20)	10 (9–15)	0.247^b^

a Fisher’s exact test.

b Mann–Whitney *U* test.

PNET, pancreatic neuroendocrine tumor; ASA, American Society of Anesthesiology; CRPOPF, clinically relevant post-operative pancreatic fistula; LN, lymph node; NA, not available.

### Comparison of long-term outcomes of patients with unresectable well-differentiated metastatic PNET treated with surgery and conservative therapy

**Figure 2.**
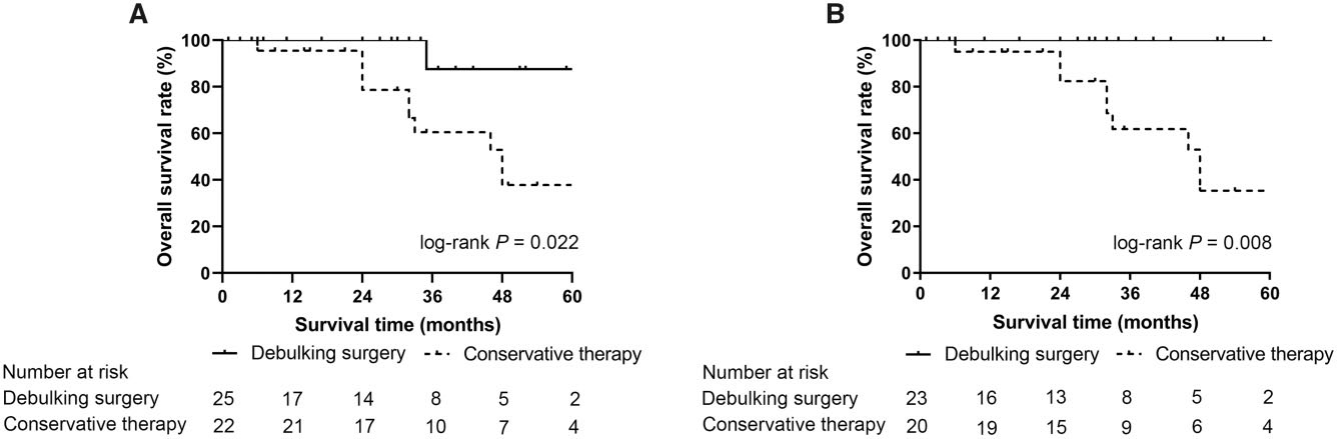
Kaplan–Meier curve for overall survival of patients with unresectable well-differentiated metastatic PNET. (A) All patients; (B) patients with G1/G2 tumors. PNET, pancreatic neuroendocrine tumor.

With the median follow-up time of 30 months, the 5-year OS rate of patients treated with debulking surgery was significantly higher than that of patients treated with conservative therapy alone (87.5% vs 37.8%, log-rank *P *=* *0.022, Figure 2A). In subgroup analysis, for patients with G1/G2 tumors, patients undergoing debulking surgery had a higher OS rate than those receiving conservative therapy (100% vs 35.3%, log-rank *P *=* *0.008, Figure 2B). Due to the small number of patients with G3 tumors, whether debulking surgery is beneficial for patients with G3 tumors could not be evaluated.

### Comparison of long-term outcomes of patients with well-differentiated metastatic PNET treated with radical surgery and debulking surgery

In order to compare the differences in OS between patients treated with debulking surgery and radical resection, 25 patients with unresectable m-PNET were assigned to the debulking surgery group while the 6 patients with resectable m-PNET treated with radical resection were assigned to the radical surgery group.

**Figure 3.**
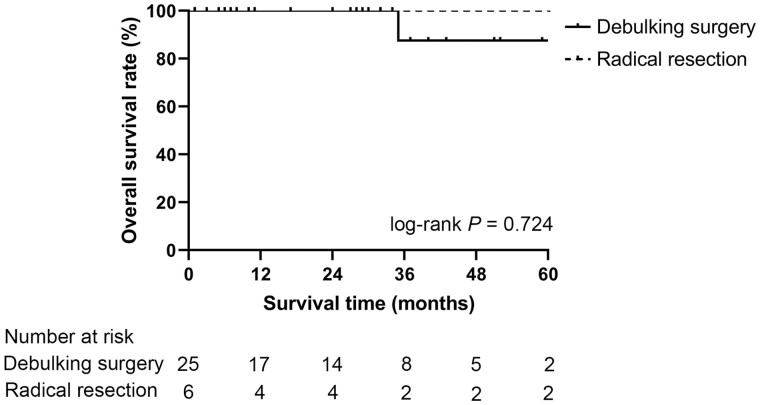
Kaplan–Meier curves for overall survival after surgery for patients with well-differentiated metastatic PNET receiving curative resection and debulking surgery. PNET, pancreatic neuroendocrine tumor.

Although the OS seemed to be superior in patients treated with radical resection, the Kaplan–Meier curve showed that there was no significant difference in the OS rate between patients undergoing radical resection and those undergoing debulking surgery (100% vs 87.5%, log-rank *P *=* *0.724, Figure 3).

### Prognostic factors of OS in patients with unresectable well-differentiated metastatic PNET

The prognostic factors evaluated by the Cox proportional hazard model are presented in Table 3. Multivariate analysis showed that debulking surgery (hazard ratio, 0.11; 95% confidence interval, 0.01–0.98; *P *=* *0.048) was an independent prognostic factor of OS in patients with unresectable well-differentiated m-PNET. However, other factors were not associated with OS in unresectable well-differentiated m-PNET, including WHO tumor grade and American Joint Committee on Cancer (AJCC) tumor node metastasis (TNM) stage.

**Table 3.** Cox proportional hazard model analysis of prognostic factors in overall survival of patients with unresectable well-differentiated metastatic PNET

	Univariate		Multivariate	
Feature	HR (95% CI)	*P*-value	HR (95% CI)	*P*-value
Debulking surgery, yes vs no	0.14 (0.02–1.06)	0.056	0.11 (0.01–0.98)	0.048
WHO grade, G3 vs G1/G2	2.14 (0.46–9.91)	0.331	0.89 (0.17–4.73)	0.895
T stage^a^, T3+T4 vs T1+T2	1.78 (0.47–6.73)	0.396	4.30 (0.95–19.37)	0.058
N stage^a^, N1 vs N0	3.66 (0.79–17.02)	0.098	1.81 (0.34–9.66)	0.487
M stage^a^, M1c vs M1a	1.77 (0.38–8.27)	0.469	1.92 (0.35–10.59)	0.454
Age^b^, <49 vs ≥49 years	1.83 (0.48–6.96)	0.375		
Sex, female vs male	0.56 (0.16–1.92)	0.356		
Diabetes, yes vs no	1.29 (0.28–6.00)	0.746		
Hypertension, yes vs no	0.44 (0.06–3.46)	0.434		
Symptom, yes vs no	1.41 (0.37–5.32)	0.614		
Functionality, yes vs no	0.82 (0.22–3.11)	0.773		
Primary tumor location, head/neck vs body/tail	0.55 (0.15–2.08)	0.379		

a American Joint Committee on Cancer 8th edition staging system.

b The median number was used as the cut-off value.

HR, hazard ratio; CI, confidence interval; WHO, World Health Organization.

## Discussion

The decision to perform surgery on patients with unresectable well-differentiated m-PNET should be weighed against the potential survival benefits with the high risk of morbidity and tumor progression. In the present study, debulking surgery with or without metastasectomy can achieve a higher 5-year OS rate than conservative therapy alone (87.5% vs 37.8%, log-rank *P *=* *0.022), which was not inferior to the outcomes in the previously reported literature [17, 18], suggesting the potential benefit of surgery for patients with unresectable well-differentiated m-PNET, regardless of the functional status of the tumor. Besides, although the OS seemed to be superior in patients treated with radical resection than in those undergoing debulking surgery, there was no significant difference in the OS between these patients. It may be related to the small number of patients undergoing radical operation in this study. However, it indicated that patients with m-PNET can still benefit from debulking surgery. Most of patients undergoing debulking surgery in our study were T1–T3 tumors (80.0%), suggesting that most of the primary tumors of m-PNET were technically resectable. In addition, the post-operative major complication (Clavien–Dindo ≥ III) rate and CRPOPF rate were comparable to the previous data [19], which indicated the safety and feasibility of pancreatectomy in removing the primary pancreatic tumor. Subgroup analysis showed that in patients with unresectable G1/G2 m-PNET, debulking surgery still achieved a better prognosis than conservative therapy. However, subgroup analysis was unable to be performed due to the small number of patients with G3 tumors. Nevertheless, we found that among patients who underwent debulking surgery, the only patient who died was a patient with a G3 tumor. In G3 tumors, the disease may still progress after debulking surgery without removing all metastases. Therefore, the therapeutic value of debulking surgery in G3 patients needs to be further investigated. In conclusion, the current study demonstrated the potential benefit of debulking surgery in patients with unresectable well-differentiated m-PNET.

The role of surgery in patients with m-PNET remains controversial. Some studies have shown that patients with m-PNET may have worse survival after surgery. Norton and colleagues [20] reported a retrospective study including 46 PNET patients, 19 patients of whom underwent pancreatectomy with concomitant hepatectomy for liver metastasis, and found that a combination of liver resection decreased 10-year disease-free survival from 66% to 25% (*P *=* *0.007). Similarly, Bettini and colleagues [21] reported a small cohort of patients with metastatic nonfunctional pancreatic neuroendocrine carcinoma, which included 19 patients who underwent pancreatectomy and 32 patients who had not undergone surgery, and found no difference in OS between the two groups (surgery: 54.3 months vs non-surgery: 39.5 months, *P *=* *0.74). However, resection can still be considered palliative therapy in patients with symptomatic disease.

In our center, the treatment strategy for unresectable metastatic pNET was discussed and determined by a multidisciplinary team consisting of a hepato-pancreato-biliary surgeon, gastroenterologist, interventional radiologist, diagnostic radiologists, and pathologist. Debulking surgery for unresectable metastatic pNET was considered in the following circumstances: (i) resectable primary pancreatic tumor; (ii) good responses of LM to systemic therapy and/or trans-hepatic arterial embolization; (iii) low LM burden or most LM burden removable, and (iv) good general condition. Otherwise, the conservative therapy was preferred.

Since the patients in this study have been enrolled since 2014, there may be differences in the treatment strategies for patients treated at early and late times. Improvement of systemic therapy such as chemotherapy and target therapy were revealed to prolong the progression-free survival of m-PNET [22–24]. In our institute, the following strategies were adopted when considering a conservative therapy regimen. For tumors with positive expression of somatostatin receptor, octreotide long-acting repeatable (LAR) could be considered a basic treatment [25]. Chemotherapy or target therapy could be considered for tumors with high Ki-67 index [22, 23, 26] whereas anti-angiogenic drugs such as sunitinib and surufatinib could be considered for tumors with abundant blood supply [22, 24]. For patients with a heavy burden of LM, TAE was commonly used. The therapeutic role of surgery combined with conservative therapy in m-PNET patients needs to be further elucidated.

Factors associated with OS have not been previously established for unresectable well-differentiated m-PNET. The current study demonstrated that debulking surgery (but not WHO grade or AJCC TNM stage) was associated with a higher OS rate in unresectable well-differentiated m-PNET. In contrast to the results of previous studies [27], there was no significant association between tumor grade and prognosis of patients in this study, which may be explained by the small number of cases of G3 tumors. Factors associated with progression and survival in patients with unresectable well-differentiated m-PNET still need to be fully elucidated.

This study has several limitations. First, this study was a single-center, retrospective study with a relatively small sample size, which may lead to biased results. Second, for patients treated with conservative therapy, the treatment regimen might vary during the treatment course because of different treatment responses, which might affect the long-term outcomes. The effectiveness of debulking surgery in patients with unresectable well-differentiated m-PNET needs to be further studied by using multicenter prospective trials.

## Conclusions

In conclusion, the current study showed that debulking surgery leads to a higher OS rate in patients with unresectable well-differentiated m-PNET than conservative therapy alone; its efficacy was close to that of radical surgery. These findings suggest that debulking surgery could provide favorable outcomes in patients with unresectable well-differentiated m-PNET.

## Supplementary Material

goad010_Supplementary_Data
