# Supplementary material for: Values of debulking surgery for unresectable well-differentiated metastatic pancreatic neuroendocrine tumors: a comparative study
Source: Gastroenterol Rep (Oxf). 2023 Mar 8;11:goad010. doi: 10.1093/gastro/goad010 (PMC9995090; doi:10.1093/gastro/goad010)
Supplement: goad010_Supplementary_Data [file goad010_supplementary_data.docx]

**Supplementary Table 1.** Comparison of liver metastasis in patients with unresectable well-differentiated metastatic pancreatic neuroendocrine tumor.

| Variable | Debulking surgery  (*n* = 25) | Conservative therapy  (*n* = 22) | *P* value^a^ |
| --- | --- | --- | --- |
| Liver metastasis type |  |  | 0.095 |
| Type II | 25 (100.0%) | 19 (86.4%) |  |
| Type III | 0 (0%) | 3 (13.6%) |  |
| Liver metastasis burden |  |  | 0.117 |
| 0-25% | 20 (80.0%) | 12 (54.5%) |  |
| 26%-50% | 4 (16.0%) | 4 (18.2%) |  |
| 51%-75% | 1 (4.0%) | 3 (13.6%) |  |
| 76%-100% | 0 (0%) | 3 (13.6%) |  |

a. Fisher exact test.

**Supplementary Table 2.** The neoadjuvant and adjuvant therapy for patients with unresectable well-differentiated metastatic pancreatic neuroendocrine tumor undergoing debulking surgery

| Treatment | Total | Somatostatin analogs | Cytotoxic chemotherapy | Molecular targeted therapy | Liver-directed therapy | Peptide receptor radionuclide therapy |
| --- | --- | --- | --- | --- | --- | --- |
| Neoadjuvant therapy | 24 (96.0%) | 19 (76.0%) | 5 (20.0%) | 6 (24.0%) | 11 (44.0%) | 0 (0%) |
| Adjuvant therapy | 25 (100%) | 24 (96.0%) | 1 (4.0%) | 6 (24.0%) | 10 (40.0%) | 1 (4.0%) |

**Supplementary Table 3.** The treatment of patients with unresectable well-differentiated metastatic pancreatic neuroendocrine tumor who received conservative therapy

| Regimen | Somatostatin analogs | Cytotoxic chemotherapy | Molecular targeted therapy | Liver-directed therapy |
| --- | --- | --- | --- | --- |
| 1-line therapy | 16 (72.7%) | 6 (27.3%) | 6 (27.3%) | 8 (36.4%) |
| 2-line therapy | 10 (45.5%) | 3 (13.6%) | 8 (36.4%) | 9 (40.9%) |
| 3-line therapy | 8 (36.4%) | 6 (27.3%) | 5 (22.7%) | 5 (22.7%) |
